# Perioperative lidocaine and dexmedetomidine intravenous infusion reduce the serum levels of NETs and biomarkers of tumor metastasis in lung cancer patients: A prospective, single-center, double-blinded, randomized clinical trial

**DOI:** 10.3389/fonc.2023.1101449

**Published:** 2023-02-24

**Authors:** Baiqing Ren, Muqiao Cheng, Chao Liu, Huiwen Zheng, Jingyue Zhang, Wei Chen, Jie Song, Jingwen Zhuang, Tianya Liu, Rui Wang, Zhiping Wang

**Affiliations:** ^1^ Department of Anesthesiology, The Affiliated Hospital of Xuzhou Medical University, Xuzhou, China; ^2^ Jiangsu Province Key Laboratory of Anesthesiology, Xuzhou Medical University, Xuzhou, China; ^3^ Department of Anesthesiology, Xinhua Hospital Affiliated to Shanghai Jiao Tong University School of Medicine, Shanghai, China; ^4^ Department of Anesthesiology, The First People’s Hospital of Changde City, Changde, China

**Keywords:** lidocaine, dexmedetomidine, tumor, metastasis, lung cancer, neutrophil extracellular traps

## Abstract

**Background:**

Neutrophil extracellular traps (NETs) can enhance the metastasis of non-small cell lung cancer (NSCLC). As biomarkers of tumor metastasis, metalloproteinases (MMPs) and vascular endothelial growth factor (VEGF) together with NETs are essential to endothelial-to-mesenchymal transition (EMT). We hypothesized that intravenous infusion of lidocaine and dexmedetomidine could reduce the production of NETs and biomarkers of tumor metastasis after video-assisted thoracic surgery (VATS) in NSCLC patients.

**Method:**

The trial included 132 NSCLC patients undergoing VATS. The patients were equally randomized to a placebo group (Group C), a lidocaine group (Group L, intravenous lidocaine 8 mg/kg/h for 15 minutes before anesthesia, 2 mg/kg/h during surgery, and 1 mg/kg/h until 24 hours after surgery), a dexmedetomidine group (Group D, intravenous dexmedetomidine 2 μg/kg/h for 15 minutes before anesthesia, 0.5 μg/kg/h during surgery, and 0.25 μg/kg/h until 24 hours after surgery), and a dexmedetomidine plus lidocaine group (Group LD, combination use of lidocaine and dexmedetomidine). The primary outcome was the production of myeloperoxidase (MPO) and citrullinated histone-3 (H3Cit), biomarkers of NETs, on postoperative day (POD) 1. MMP-3, MMP-9, and VEGF-α, as biomarkers of tumor metastasis, were also evaluated on POD 1.

**Results:**

The baseline patient characteristics and perioperative data did not differ between the study groups. MPO was significantly decreased in Groups L, D, and LD (-197.08 ± 34.01, -137.37 ± 32.41, and -189.45 ± 33.73 U/ml, *P*<0.001, respectively) compared with Group C (-106.51 ± 25.44 U/ml). H3Cit was also lessened in Groups L, D, and LD (-49.51 ± 9.11, -34.80 ± 10.37, and -51.82 ± 8.98 ng/ml, *P*<0.001, respectively) compared with Group C (-24.73 ± 7.65 ng/ml). Lidocaine and dexmedetomidine also reduced MMP-3 (-69.08 ± 13.22, -52.84 ± 13.78, -85.34 ± 12.59 *vs.* -40.55 ± 10.71 ng/ml in Group L, D, LD *vs.* Group C, *P*<0.001, respectively), MMP-9 (-8.46 ± 1.68, -6.07 ± 1.82, -9.67 ± 1.43 *vs.* -4.28 ± 1.29 ng/ml in Group L, D, LD *vs.* Group C, P<0.001, respectively), and VEGF-α (-95.55 ± 22.53, -71.65 ± 18.77, -104.89 ± 15.49 *vs.* -51.73 ± 16.27 pg/ml in Group L, D, LD *vs.* Group C, P<0.001, respectively) on POD 1.

**Conclusion:**

In NSCLC patients, continuous perioperative intravenous infusion of lidocaine and dexmedetomidine significantly reduced the production of NETs and tumor metastasis biomarkers on POD 1. Meanwhile, it also decreased inflammation, protected cellular immune function, reduced pain and opioid consumption, and improved the quality of postoperative recovery.

**Clinical trial registration:**

chictr.org.cn, identifier: 187049.

## Foundations

1. National Natural Science Foundation of China, 82270059;
2. Jiangsu Provincial Health Commission Medical Research Key Project, K2019003;
3. Jiangsu Natural Science Foundation, BK20221222; China Primary Health Care Foundation, YLGX-MZ-2022004;
4. The foundation of Xuzhou Science and Technology Department, KC21154.

## 1 Introduction

Non-small cell lung cancer (NSCLC) is the leading cause of cancer death worldwide due mainly to recurrence and metastasis (1). Surgery as the optimal curative option for early-stage NSCLC patients could paradoxically increase the development of metastases (2). Perioperative inflammation and sympathetic nervous system (SNS) activation are closely related to tumor metastatic progression (3). Anesthesia interventions are an essential part of the perioperative period and have been shown to be a potential way to decrease tumor metastasis risk by reducing stress and inflammation levels and preserving immune system function in NSCLC patients (4).

Neutrophil extracellular traps (NETs) are sticky substances containing DNA and various proteases released during the apoptosis of neutrophils (5). NETs have emerged recently as a new biomarker of tumor metastasis (6). NETs can not only enhance metastases of NSCLC (7) or other cancers (8, 9) but also awaken dormant cancer cells (10). NETs, along with metalloproteinases (MMP) and vascular endothelial growth factor (VEGF), promote vascular leakage and endothelial-to-mesenchymal transition (EMT) (11).

The amide local anesthetic lidocaine (Lido) has anti-inflammatory effects and has been widely used clinically. Recent studies proved that perioperative intravenous administration of lidocaine could reduce NETs in pancreatic cancer patients (12) and peripheral circulation levels of VEGF in breast cancer patients (13). Dexmedetomidine (Dex), as a highly selective α2-adrenergic receptor agonist, plays an important role in anesthesia and has been commonly used during thoracic surgical anesthesia. It has been demonstrated that preoperative administration of Dex can not only improve intraoperative oxygenation, lung mechanics, and the quality of postoperative recovery (14, 15) but also alleviate SNS activation and the inflammatory response, immunosuppression, and postoperative pain (16). It is not known whether Lido and Dex minimize the production of perioperative NETs in NSCLC patients.

The primary purpose of our study was to investigate whether the continuous intravenous infusion of Lido and Dex during the perioperative period could reduce the production of NETs in patients with NSCLC. Several studies have shown that it is feasible to detect markers of NET formation in serum (e.g., myeloperoxidase (MPO) and citrullinated histone-3 (H3Cit)) (13, 17), so we selected MPO and H3Cit as alternative indicators of NET formation in our study. This study will evaluate tumor metastasis biomarkers (VEGF-A, MMP-3, MMP-9), inflammatory factors, immune function and clinical outcomes.

## 2 Materials and methods

This study was a prospective, single-center, randomized, double-blinded clinical trial. The protocol was approved by the Ethics Committee of the Affiliated Hospital of Xuzhou Medical University (XYFY2022-KL254-01). Before the inclusion of the first patient, the trial was registered at the Chinese Clinical Trial Registry (ChiCTR2100050796; leading researcher: Zhiping Wang). This study was carried out between July 22, 2022, and October 2, 2022. Written informed consent was obtained from patients before they participated in the study.

### 2.1 Patients

Inclusion criteria were as follows: 18 to 80 years of age, American Society of Anesthesiologists (ASA) physical status I, II, or III, scheduled for elective video-assisted thoracic surgery, consent to receive postoperative patient-controlled analgesia (PCA), early-stage lung cancer without distant dissemination (clinical stage ≤ II), and willing to accept follow-up. The preoperative diagnosis of early-stage lung cancer was based on assessment by thoracic surgeons and findings from computed tomography and positron emission tomography-computed tomography. The final diagnosis of early-stage lung cancer depended on the results of intraoperative rapid frozen pathology and postoperative routine pathological examination. The exclusion criteria were preoperative adjuvant chemotherapy or radiotherapy, pregnancy, previous treatment with beta-blockers, hepatic or renal insufficiency or dialysis, obstructive pneumonia or other acute infections not well controlled, opioid abuse, mental health problems or cognitive impairment, history of epilepsy and taking antiepileptic drugs, chronic inflammatory or autoimmune disease and glucocorticoids or other inflammatory immunosuppressive therapy taken within the past month. Patients were removed from the trial if treated with glucocorticoids perioperatively or transferred to the ICU.

### 2.2 Randomization, concealment, and blinding

Patients were randomly assigned to one of the four groups using IBM SPSS 24.0 software (SPSS Inc., IBM, Chicago, IL, USA) in a 1:1:1:1 ratio by investigators not involved in the trial. The experimental groups were the placebo group (C), the placebo plus Lido group (L), the DEX plus placebo group (D), and the DEX plus Lido group (LD).

The pharmacy personnel performed the blinding by writing a random number in a sealed and opaque envelope. The medication was applied according to the protocol in the envelope. The drugs handed over to the anesthesiologist were labeled only with the method of administration. The pumping rate was set consistently at a rate of 0.4 ml/kg/h 15 minutes before anesthesia and 0.1 ml/kg/h after induction of anesthesia. Specifically, Lido was pumped at 8 mg/kg/h for 15 minutes before anesthesia, 2 mg/kg/h during surgery, and 1 mg/kg/h until 24 hours after surgery. Dex was pumped at 2 μg/kg/h for 15 minutes before anesthesia, 0.5 μg/kg/h during surgery, and 0.25 μg/kg/h until 24 hours after surgery.

The researcher was blinded to the grouping of patients. All trial personnel received standardized training, and the same surgeons performed all procedures.

### 2.3 Protocol and intervention

All patients routinely fasted for 6 to 8 hours. Once the patient arrived in the operating room, the anesthesiologist established peripheral venous access and performed a standardized monitoring process. Monitored parameters and procedures of monitoring included noninvasive blood pressure (NIBP), ECG, pulse oximetry, invasive blood pressure (IBP), bispectral index (BIS, VISTA™ Monitoring System, Aspect Medical Systems Inc., Norwood, MA, USA) and a train-of-four stimulation (TOF) (BeneVision N17/N15 OR, Mindray Medical International Co., Guangdong, China).

A standardized anesthesia process was implemented for each patient. The intervention described above was started before the induction of anesthesia. General anesthesia was induced using a combination of sufentanil 0.3-0.6 μg/kg, etomidate 0.2-0.4 mg/kg, and cis-atracurium 0.15-0.25 mg/kg. Tracheal intubation was performed under visual laryngoscopy after induction. The ventilator parameters were set as follows: tidal volume 6–8 mL/kg; respiratory rate 12–16 times/min; ratio of aspiration to expiration 1:1.5; fraction of inspired oxygen 60-100%; and positive end-expiratory pressure 0-6 cmH2O. Anesthesia was maintained with propofol 2-10 mg/kg/h and remifentanil 0.1-0.8 μg/kg/min, with propofol dose adjustment based on BIS values (fluctuating between 40-50) and remifentanil adjustment based on intraoperative hemodynamic parameters (fluctuating ±10% from preoperative level); cisatracurium was infused intraoperatively at 1-2 μg/kg/min.

At the end of the surgery, the lungs were bilaterally aspirated, and the lung on the operative side was treated with pulmonary resuscitation (repeated puffing at a pressure of 15-25 cmH_2_O until no bubbles were visible from the negative pressure drainage device). All anesthetic drugs were discontinued except for the experimental drugs. Then, the patients were transferred to the postanesthesia care unit for further observation and subsequently transferred back to the surgical ward.

All patients were treated with an electronic PCA pump (sufentanil 100 μg plus tropisetron 10 mg) for postoperative analgesia. The PCA background infusion rate was initially set at 2 ml/h, and an additional 0.5 ml of solution was given per compression. The PCA adjustment was performed by trained thoracic nursing staff. PCA was suspended at 72 hours postoperatively.

### 2.4 Collection of blood samples

The patient’s preoperative blood samples (5 ml) were collected before anesthesia was performed, and postoperative blood samples were collected 24 hours after the operation. Blood samples were obtained from the patient’s median cubital vein and injected into a procoagulation tube. After being left to coagulate for 1 hour at room temperature, the blood was centrifuged at 3000 rpm for 15 minutes. Subsequently, 2 ml of serum was acquired from the upper layer of the blood and transferred to sterile EP tubes, which were then stored at -80°C in a refrigerator for further enzyme-linked immunosorbent assay (ELISA) experiments.

### 2.5 Neutrophil extracellular trapping and other tests

MPO, H3Cit, MMP-3, MMP-9, and VEGF-α were measured using commercially available ELISA kits (Shanghai Lanpai Biotechnology Co.). All test procedures were carried out strictly according to the ELISA kits’ instructions. Due to financial constraints, repeat measurements were not performed. Inflammatory factors, including IL-1β, IL-2, IL-4, IL-6, IFN-γ and TNF-α, were tested. The numbers of neutrophils, lymphocytes, and lymphocyte subsets, including the percentages of CD4+ T cells, CD8+ T cells, and NK cells, were also investigated perioperatively. The hospital laboratory department measured inflammatory factor levels, blood cell counts, and lymphocyte subsets. Blood sample collection was performed by the staff responsible for blood sample collection in this study. We also investigated the Th1:Th2 balance by evaluating the IFN-γ:IL-4 ratio.

### 2.6 Outcome measures

The primary outcomes were the levels of NET-specific markers (MPO and H3Cit) on postoperative day (POD) 1. Secondary outcomes included tumor metastasis biomarkers (VEGF-α, MMP-3, MMP-9), inflammatory factors (IL-1β, IL-2, IL-4, IL-6, IFN-γ, and TNF-α), immune function (the counts of macrophages, neutrophils, and lymphocytes, the percentages of CD4+ T cells, CD8+ T cells, NK cells, the ratio of CD4+:CD8+ T cells, and the ratio of IFN-γ:IL-4), intraoperative remifentanil consumption, time to the first use of PCA, time to the first exhaust and defecation, drainage removal time, bed-leaving time, length of hospital stay (LOS), sufentanil consumption in the first 3 days postoperatively, VAS scores at rest and activity during the 3 days after surgery, postoperative quality of recovery based on the 40-item Quality of Recovery (QoR-40) questionnaire administered during the first 3 days after surgery, incidence of postoperative pulmonary complications (PPCs) during hospitalization, all-cause mortality within 30 days postoperatively, and rate of readmission due to complications within 30 days postoperatively.

### 2.7 Sample-size calculation

The sample size was calculated using PASS software (version 15.0.5, NCSS, LCC, USA). The difference in serum levels of MPO before and after surgery, the measurement of which required a larger sample size than H3Cit testing, was chosen for calculation based on the results of the pre-experiment ELISA. The results showed that the differences in MPO in the C group, L group, D group, and LD group were -86.9 ± 22.26, -177.8 ± 27.04, -137.2 ± 16.32 and -218.4 ± 20.16 U/L, respectively. All pairwise types in multiple comparisons in PASS software were selected for sample size calculation. The minimum detectable difference of the mean from the results was 40.6 U/L, and a maximum standard deviation of 27.04 U/L was chosen. Assuming a type I error of 0.05 and a type II error of 0.1, 30 patients per group were needed to ensure 90% power. Considering the possibility of drop-out, 33 patients were eventually required for each group.

### 2.8 Statistical analysis

All statistical analyses were conducted using IBM SPSS 24.0 software, and plots were generated using GraphPad Prism 8 (GraphPad Software, LLC, San Diego, CA, USA). The normality and homogeneity of the data were assessed using the Kolmogorov−Smirnov test, and Levene’s test was used for continuous data. Normally distributed data were expressed as the mean ± standard deviation and examined using one-way ANOVA. If the homogeneity of variance was not achieved, then the Kruskal−Wallis test was performed.

A generalized linear model (GLM) was applied to test the differences in metastasis biomarkers, inflammation factors, peripheral blood cells, and cellular immune functions. The preoperative level was chosen as a covariable during analysis. The nonnormally distributed data are presented as the median (IQR) and were tested with the Kruskal−Wallis test. Categorical variables are reported as frequencies (%) and were analyzed using Fisher’s exact test.

Repeatedly measured data (VAS scores and QoR-40 scores) were analyzed using a generalized estimating equation (GEE) with a robust estimation as the covariance matrix. An unstructured working correlation matrix was selected during the GEE process. The Kaplan−Meier test was applied to describe and analyze the differences in time to the first use of PCA, time to the first exhaustion and defecation, drainage removal time, and bed-leaving time. The Bonferroni method was applied to correct *P value*s when multiple comparisons were conducted. The effect size expressed as a partial η^2^ was also calculated.

Tumor metastasis biomarkers, inflammatory factors, and cellular immune function were included as independent variables, and NETs were taken as dependent variables in the multifactorial regression. The stepwise method was chosen when the probability corresponding to the *F value* of the independent variable was included if <0.05 and excluded if >0.10. All statistical analyses were two-tailed tests, and a *P value* < 0.05 was recognized as indicative of significant difference.

## 3 Results

**Figure 1:**
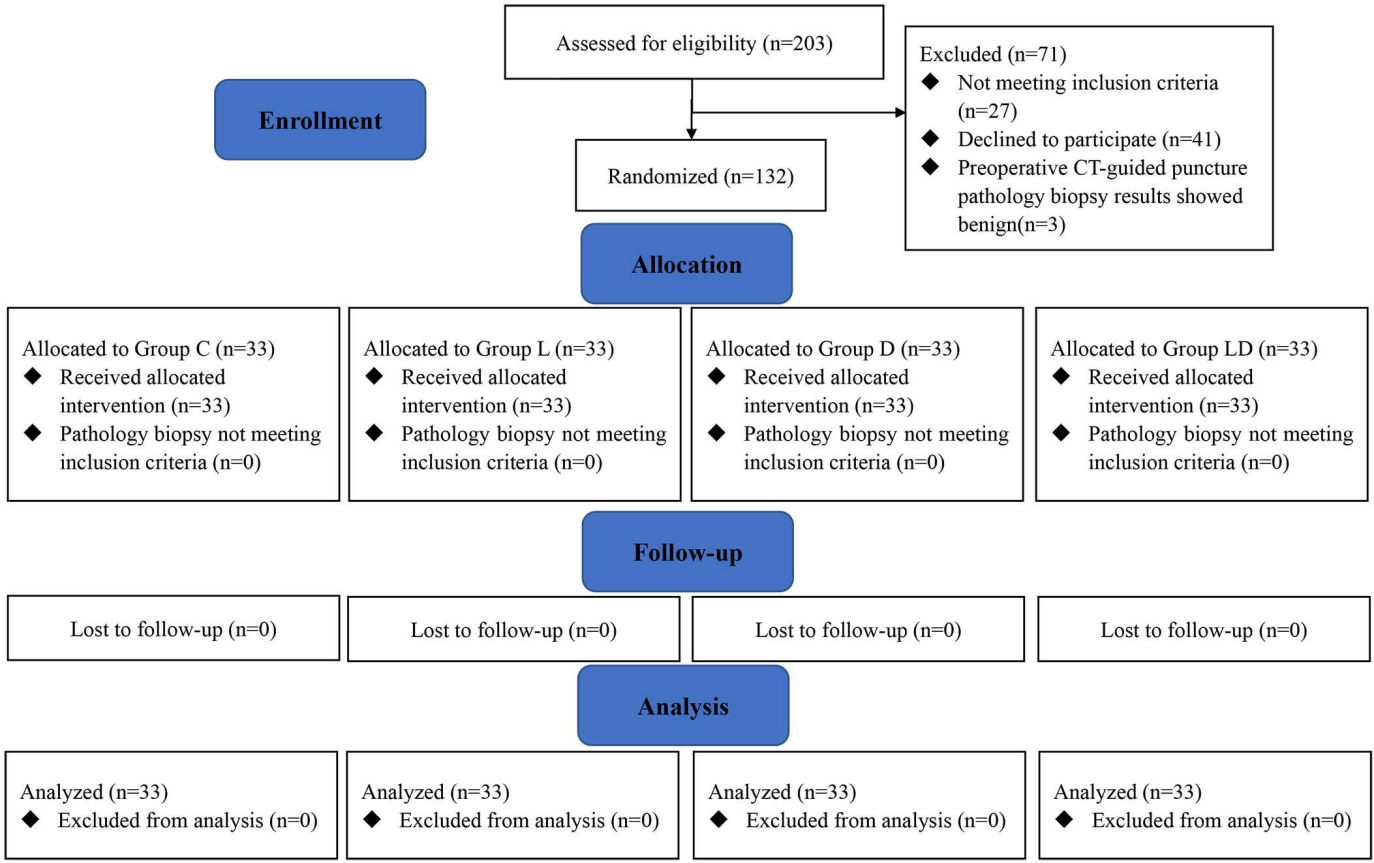
Study flow diagram based on the Consolidated Standards of Reporting Trials (CONSORT) statement. Group C, placebo group; Group L, placebo plus lidocaine group; Group L, dexmedetomidine plus placebo group; Group LD, dexmedetomidine plus lidocaine group.

Between July 22, 2002, and October 2, 2022, 203 subjects were enrolled, and 132 were randomized into four groups (**Figure 1**). Eventually, 33 patients from each group were included in the final analysis. There was no loss to follow-up one month after surgery. The last patient was admitted on October 2.

### 3.1 Participants

The baseline patient demographic and clinical characteristics in the four groups were comparable (**Table 1**). The mean arterial pressure remained constant during anesthesia and surgery (**Table 2**). There was no dose difference between perioperative Lido and Dex (*P*=0.147) in the drug-applying group (**Table 2**). Other intraoperative and surgical-related characteristics were identical among the four groups, except for the differences in intraoperative remifentanil dosage (*P*<0.001) and propofol dosage (*P*=0. 008) (**Table 2**).

**Table 1:** Baseline demographic and clinical characteristics.

Variables	Group C (n=33)	Group L (n=33)	Group D (n=33)	Group LD (n=33)	*P*-value
Age, y	60.67 ± 7.90	60.00 ± 10.54	57.21 ± 10.19	60.48 ± 8.42	0.403
BMI, kg/m^2^	24.93 ± 3.59	23.04 ± 2.87	24.16 ± 2.73	23.71 ± 2.34	0.068
Female sex (female)	17(51.5)	16(48.5)	16(48.5)	17(51.5)	1.000
ASA physical status					0.309
II	29(87.9)	30(90.9)	33(100)	31(93.9)	
III	4(12.1)	3(9.1)	0(0)	2(6.1)	
Mini-Mental State Examination,	26.12 ± 1.56	27.00 ± 1.52	26.52 ± 1.72	26.82 ± 1.36	0.110
Age-adjusted Charlson Comorbidity Index					0.780
≤2	1(3.0)	1(3.0)	0(0)	1(3.0)	
3	2(6.1)	2(6.1)	4(12.1)	0(0)	
4	3(9.1)	4(12.1)	5(15.2)	5(15.2)	
≥5	27(81.8)	26(78.8)	24(72.7)	27(81.8)	
Catalonia score	54.79 ± 8.49	56.94 ± 8.17	54.18 ± 7.72	54.67 ± 7.79	0.516
Catalonia estimated risk level					1.000
Middle risk	3(9.1)	3(9.1)	4(12.1)	3(9.1)	
High risk	30(90.9)	30(90.9)	29(87.9)	30(90.9)	
Arterial blood gas analysis before surgery					
SpO2(%)	94.15 ± 1.62	93.97 ± 1.70	94.55 ± 1.89	94.64 ± 1.83	0.366
K+	3.68 ± 0.36	3.65 ± 0.50	3.52 ± 0.35	3.46 ± 0.25	0.052
Na+	138.79 ± 2.92	138.52 ± 2.90	138.97 ± 3.39	138.76 ± 2.83	0.944
Blood glucose (mmol/L)	6.47 ± 1.54	6.50 ± 1.53	6.46 ± 1.54	5.98 ± 1.43	0.440

The data are the mean ± SD, n (%), or median (interquartile range). Group C, placebo group; Group L, placebo plus lidocaine group; Group L, dexmedetomidine plus placebo group; Group LD, dexmedetomidine plus lidocaine group. BMI, Body mass index; ASA, American Society of Anesthesiologists. *P<0.05 was statistically significant.

**Table 2:** Surgical and intraoperative characteristics.

Variables	Group C (n=33)	Group L (n=33)	Group D (n=33)	Group LD (n=33)	P-value
Duration of surgery (hours)	2.82 ± 0.25	2.94 ± 0.28	2.92 ± 0.28	2.96 ± 0.27	0.186
Type of surgery					0.544
Lobectomy	17(51.5)	22(66.7)	23(69.7)	19(57.6)	
Segmentectomy	4(12.1)	1(3.0)	2(6.1)	1(3.0)	
Wedge resection	12(36.4)	10(30.3)	8(24.2)	13(39.4)	
Stage					0.833
Tis	8(24.2)	8(24.2)	6(18.2)	10(30.3)	
I	20(60.6)	19(57.6)	19(57.6)	15(45.5)	
II	5(15.2)	6(18.2)	8(24.2)	8(24.2)	
Tumor size(cm)	1.26 ± 1.02	1.72 ± 1.31	1.85 ± 1.25	1.41 ± 1.12	0.161
Tumor location					0.353
Right upper lobe	15(45.5)	9(27.3)	12(36.4)	10(30.3)	
Right middle lobe	2(6.1)	0	2(6.1)	2(6.1)	
Right lower lobe	5(15.2)	10(30.3)	6(18.2)	3(9.1)	
Left upper lobe	9(27.3)	9(27.3)	9(27.3)	9(27.3)	
Left lower lobe	2(6.1)	5(15.2)	4(12.1)	9(27.3)	
Intraoperative remifentanil (μg)	3329.39 ± 785.91	2376.06 ± 616.81†	3362.12 ± 827.90‡	1946.67 ± 495.38†§	<0.001*
Intraoperative crystalloids (L)	2095.76 ± 431.99	2056.06 ± 460.556	2196.06 ± 435.65	2060.91 ± 342.42	0.501
Intraoperative atracurium (mg)	20.17 ± 4.65	20.83 ± 4.91	21.82 ± 4.85	20.49 ± 3.57	0.486
Intraoperative propofol (mg)	986.67 ± 206.38	848.18 ± 172.74†	923.64 ± 193.58	848.48 ± 180.16†	0.008*
Intraoperative BIS	62.55 ± 2.17	62.76 ± 1.74	62.21 ± 1.71	62.79 ± 1.69	0.558
Intraoperative HR	68.10 ± 10.76	65.65 ± 9.27	64.48 ± 10.88	61.72 ± 9.61	0.086
Intraoperative MAP (mmHg)	82.75 ± 6.79	84.65 ± 9.13	82.22 ± 8.48	87.45 ± 7.37	0.038*
Estimated blood loss (ml)	95.45 ± 14.60	91.21 ± 14.53	96.36 ± 13.65	93.03 ± 12.37	0.421
Estimated urine volume (ml)	602.42 ± 359.87	583.94 ± 361.61	713.03 ± 343.34	558.18 ± 297.61	0.272
Total lidocaine administration (mg)	0	2080.11 ± 322.50	0	2054.37 ± 267.53	0.725^a^
Total dexmedetomidine administration (μg)	0	0	541.28 ± 85.13	513.60 ± 66.89	0.147^a^

The data are the mean ± SD, n (%). Group C, placebo group; Group L, placebo plus lidocaine group; Group L, dexmedetomidine plus placebo group; Group LD, dexmedetomidine plus lidocaine group. BIS, Bispectral index; HR, Heart rate; MAP, Mean arterial pressure. ^a^Analysis was performed by two independent sample t-tests. *P<0.05 was statistically significant.

†Compared with the placebo group(C), the difference was statistically significant.

‡Compared with the placebo plus lidocaine group (L), the difference was statistically significant.

§Compared with the dexmedetomidine plus placebo group (D), the difference was statistically significant.

### 3.2 Primary outcomes

**Figure 2:**
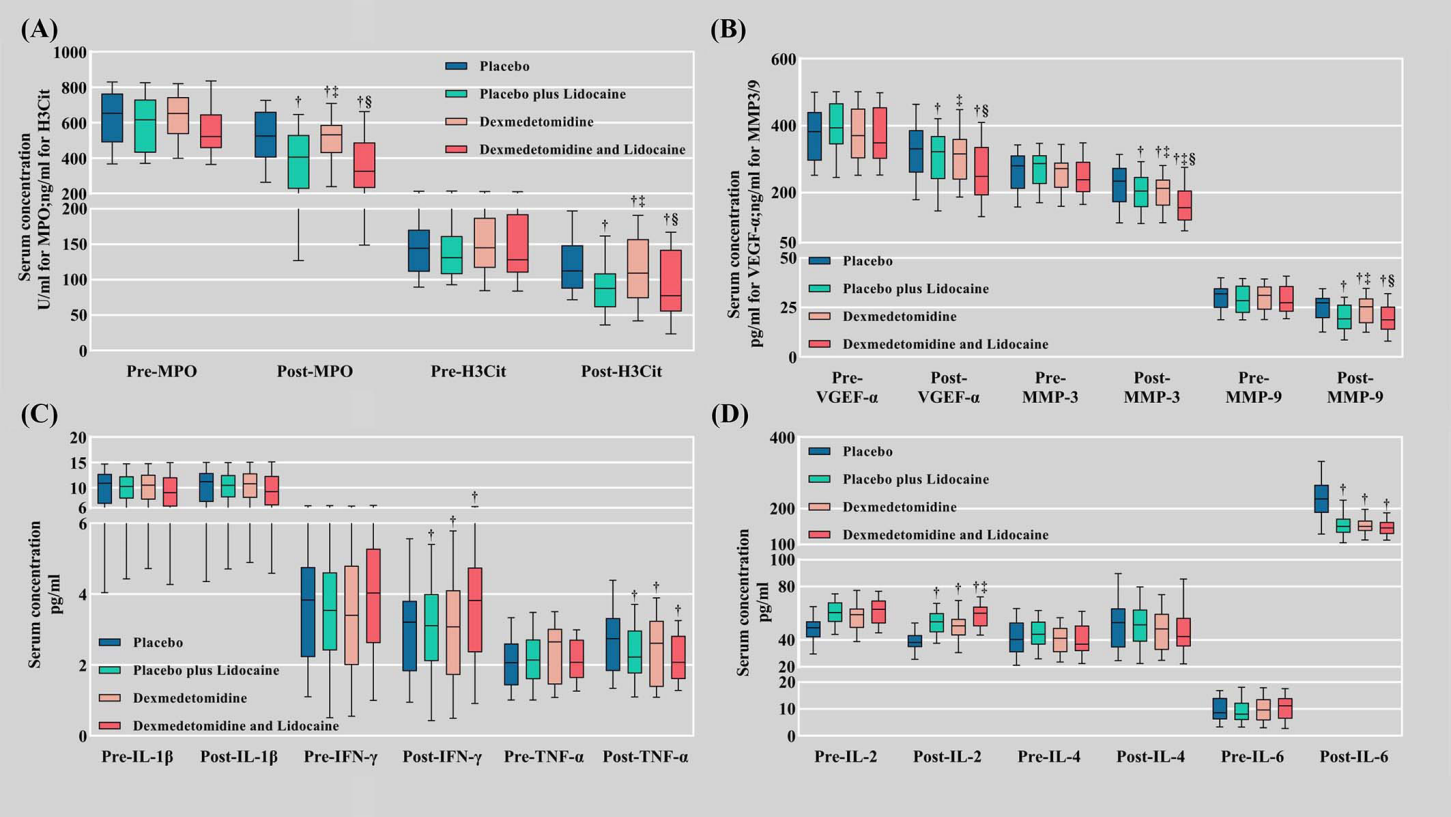
Serum biomarker levels. **(A)** Biomarkers of NET formation (MPO and H3Cit); **(B)** Metastasis biomarkers (VEGF-α, MMP-3, and MMP-9); **(C)** Inflammatory factors (IL-1β, IFN-γ, TNF-α); **(D)** Inflammatory factors (IL-2, IL-4, and IL-6). Pre, preoperative; Post, postoperative; NETs, neutrophil extracellular traps; MPO, myeloperoxidase; H3Cit, citrullinated histone 3; VEGF-A, vascular endothelial growth factor-A; MMP-3, matrix metalloproteinase-3; MMP-9, matrix metalloproteinase-9. † Compared with the placebo group (C), the difference was statistically significant. ‡ Compared with the placebo plus lidocaine group (L), the difference was statistically significant. § Compared with the dexmedetomidine plus placebo group (D), the difference was statistically significant.

The biomarkers of NETs differed among the four groups. All the results were obtained by subtracting the preoperative level from the postoperative level. MPO was significantly decreased in Groups L, D, and LD (-197.08 ± 34.01, -137.37 ± 32.41, and -189.45 ± 33.73 U/ml, P<0.001, respectively) compared with Group C (-106.51 ± 25.44 U/ml). H3Cit was also lessened in Groups L, D, and LD (-49.51 ± 9.11, -34.80 ± 10.37, and -51.82 ± 8.98 ng/ml, P<0.001, respectively) compared with Group C (-24.73 ± 7.65 ng/ml) (**Table 3**, **Figure 2A**).

**Table 3:** Primary outcome and secondary outcomes.

Variables	Group C (n=33)	Group L (n=33)	Group D (n=33)	Group LD (n=33)	*P*-value	Effect size
Primary Outcome						
NETs biomarkers Dif.						
MPO (U/ml)	-106.51 ± 25.44	-197.08 ± 34.01†	-137.37 ± 32.41†‡	-189.45 ± 33.73†§	<0.001*	0.576
H3Cit (ng/ml)	-24.73 ± 7.65	-49.51 ± 9.11†	-34.80 ± 10.37†‡	-51.82 ± 8.98†§	<0.001*	0.604
Secondary Outcomes						
Metastasis biomarkers Dif.						
VEGF-α (pg/ml)	-51.73 ± 16.27	-95.55 ± 22.53†	-71.65 ± 18.77†‡	-104.89 ± 15.49†§	<0.001^a^*	0.567
MMP-3 (ng/ml)	-40.55 ± 10.71	-69.08 ± 13.22†	-52.84 ± 13.78†‡	-85.34 ± 12.59†‡§	<0.001*	0.650
MMP-9 (ng/ml)	-4.28 ± 1.29	-8.46 ± 1.68†	-6.07 ± 1.82†‡	-9.67 ± 1.43†§	<0.001^a^*	0.649
Inflammatory factors Dif.						
IL-1β (pg/ml)	0.25 ± 0.09	0.22 ± 0.07	0.21 ± 0.10	0.19 ± 0.09	0.062	0.056
IL-2 (pg/ml)	-9.40 ± 2.94	-8.19 ± 2.91†	-7.06 ± 3.34†	-3.55 ± 2.73†‡	<0.001*	0.565
IL-4 (pg/ml)	9.77 ± 8.08	6.82 ± 7.19	6.88 ± 6.40	6.06 ± 6.30	0.105	0.047
IL-6 (pg/ml)	218.86 ± 50.83	144.52 ± 24.00†	143.40 ± 21.61†	136.43 ± 19.86†	<0.001*	0.551
IFN-γ (pg/ml)	-0.71 ± 0.36	-0.45 ± 0.23†	-0.43 ± 0.30†	-0.39 ± 0.29†	<0.001*	0.329
TNF-α (pg/ml)	0.49 ± 0.24	0.12 ± 0.22†	0.07 ± 0.23†	0.08 ± 0.17†	<0.001*	0.442
Peripheral blood immune cells Dif.						
Macrophages (×10^9^/ml)	0.19 ± 0.19	0.09 ± 0.18	0.13 ± 0.17	0.10 ± 0.14	0.112	0.046
Neutrophils (×10^9^/ml)	7.33 ± 0.97	7.33 ± 0.83	7.44 ± 0.94	7.25 ± 0.88	0.893	0.005
Lymphocytes (×10^9^/ml)	-0.68 ± 0.32	-0.32 ± 0.13†	-0.28 ± 0.16†	-0.28 ± 0.12†	<0.001*	0.424
Assay of cellular immune function Dif.						
CD4+T cells (%)	-5.12 ± 1.09	-3.60 ± 0.98†	-3.31 ± 1.19†	-3.55 ± 1.26†	<0.001*	0.293
CD8+T cells (%)	4.37 ± 3.23	3.62 ± 2.98	4.65 ± 2.79	3.81 ± 2.78	0.475	0.019
NK cells (%)	-4.46 ± 1.57	-3.06 ± 1.68†	-3.15 ± 1.22†	-1.99 ± 1.29†‡§	<0.001*	0.275
CD4+:CD8+ T cells ratio	-0.49 ± 0.25	-0.37 ± 0.15	-0.42 ± 0.16	-0.40 ± 0.18	0.053	0.059
IFN-γ:IL-4 ratio	-0.03 ± 0.02	-0.02 ± 0.01†	-0.02 ± 0.02†	-0.02 ± 0.02†	<0.001*	0.122

The data were the mean ± SD. Group C, placebo group; Group L, placebo plus lidocaine group; Group L, dexmedetomidine plus placebo group; Group LD, dexmedetomidine plus lidocaine group. Dif., differences; NETs, neutrophil extracellular traps; MPO, myeloperoxidase; H3Cit, citrullinated histone 3; VEGF-A, vascular endothelial growth factor-A; MMP-3, matrix metalloproteinase-3; MMP-9, matrix metalloproteinase-9. A generalized linear model (GLM) was applied to test the differences of biomarkers of NETs and metastasis, inflammatory factors, peripheral blood cells, and cellular immune function, and preoperative levels were chosen as covariable during analysis. Bonferroni correction was applied during multiple comparisons. The effect size was expressed as a partial η squared. *P<0.05 was statistically significant. All differences were obtained by subtracting the preoperative level from the postoperative level.

†Compared with the placebo group(C), the difference was statistically significant.

‡Compared with the placebo plus lidocaine group (L), the difference was statistically significant.

§Compared with the dexmedetomidine plus placebo group (D), the difference was statistically significant.

### 3.3 Secondary outcomes

#### 3.3.1 Biomarkers of tumor metastasis

On POD 1, serological biomarkers of tumor metastasis MMP-3 (-69.08 ± 13.22, -52.84 ± 13.78, -85.34 ± 12.59 ng/ml *vs*. -40.55 ± 10.71 ng/ml in Group L, D, LD *vs*. Group C, P<0.001, respectively), MMP-9 (-8.46 ± 1.68, -6.07 ± 1.82, -9.67 ± 1.43 ng/ml in Group L, D, LD *vs*. Group C, P<0.001, respectively), and VEGF-α (-95.55 ± 22.53, -71.65 ± 18.77, -104.89 ± 15.49 *vs*. -51.73 ± 16.27 pg/ml in Group L, D, LD *vs*. Group C, P<0.001, respectively) were also decreased in the Lido and Dex groups (**Table 3**, **Figure 2B**).

#### 3.3.2 Inflammatory factors

Among the inflammatory indicators, IL-1β and IL-4 did not differ significantly between the four groups at 24 hours postoperatively. The postoperative elevation of IL-6 (*P*<0.001) and TNF-α (*P*<0.001) was markedly suppressed in the pharmacological intervention groups (L, D, and LD) compared to group C. In contrast, the postoperative decrease in IL-2 (*P*<0.001) and IFN-γ (*P*<0.001) was dramatically retarded in the pharmacological intervention group compared to group C (**Table 3**, **Figures 2C, D**).

#### 3.3.3 Cellular immune function

**Figure 3:**
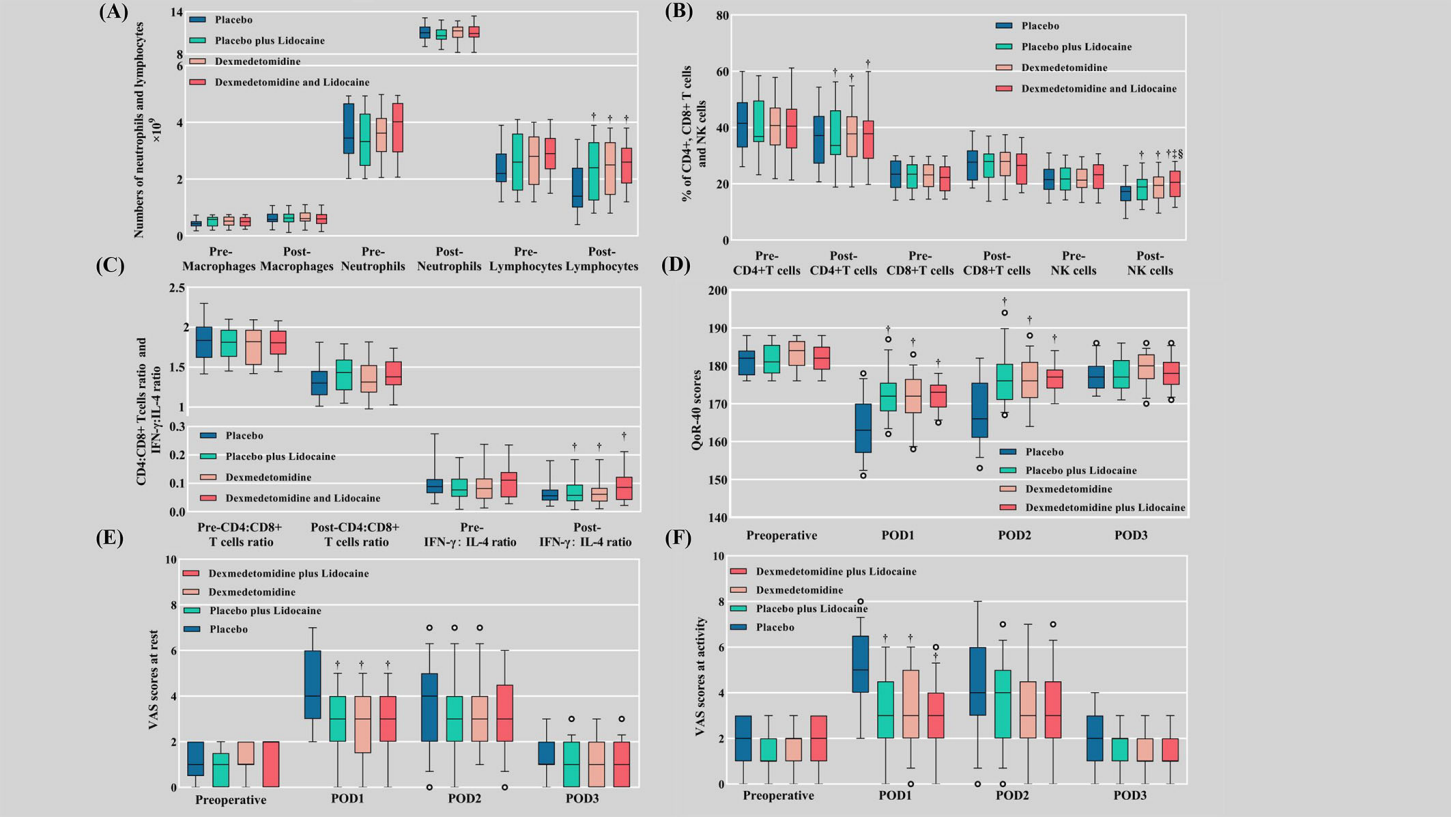
Cellular immune function, VAS scores, and QoR-40 scores. **(A)** Numbers of macrophages, neutrophils, and lymphocytes; **(B)** Percentages of CD4+ T cells, CD8+ T cells, and NK cells; **(C)** The ratios of CD4+:CD8+ T cells and IFN-γ:IL-4; **(D)** QoR-40 scores; **(E)** VAS scores at rest; **(F)** VAS scores at activity. Pre, preoperative; Post, postoperative; VAS, visual analog scale; QoR-40, Quality of Recovery 40; POD, postoperative day. † Compared with the placebo group (C), the difference was statistically significant. ‡ Compared with the placebo plus lidocaine group (L), the difference was statistically significant. § Compared with the dexmedetomidine plus placebo group (D), the difference was statistically significant.

Postoperative neutrophil counts showed no difference in elevation between groups, while lymphocyte counts (*P*<0.001) decreased in the pharmacological intervention groups. Postoperative macrophage elevation was not significantly different between groups (**Figure 3A**). The postoperative decrease in the percentage of NK cells was slight in groups L, D, and LD versus C (*P*<0.001) (**Table 3**) (**Figure 3B**). The trend of CD4+ T-cell changes (*P*<0.001) mirrored the pattern of lymphocyte counts (**Figure 3B**), whereas an examination of the numbers of CD8+ T cells (**Figure 3B**) and the ratio of CD4+:CD8+ T cells (**Figure 3C**) revealed no significant difference between groups. The postoperative decrease in the IFN-γ:IL-4 ratio in the pharmacological intervention group was markedly slowed, which was significantly different from that in group C (*P*<0.001) (**Figure 3C**).

#### 3.3.4 VAS and QoR-40

The consumption of sufentanil at 3 days postoperatively was the lowest in the LD group, followed by the L, D, and C groups sequentially (*P*<0.001). There were no differences in preoperative VAS scores and QoR-40 scores between groups (**Figures 3D–F**). Both at-rest VAS scores (*P*<0.001) and at-activity VAS scores (*P*<0.001) were inferior in the pharmacological intervention group compared to group C at POD 1, while there was no discrepancy between the four groups at POD 2 and POD 3. QoR-40 scores were better in the pharmacological intervention group than in group C at POD 1 (*P*<0.001) and POD 2 (*P*<0.001), while there was no difference between the four groups at POD 3 (**Supplementary Table 1**).

#### 3.3.5 Other postoperative observations

**Figure 4:**
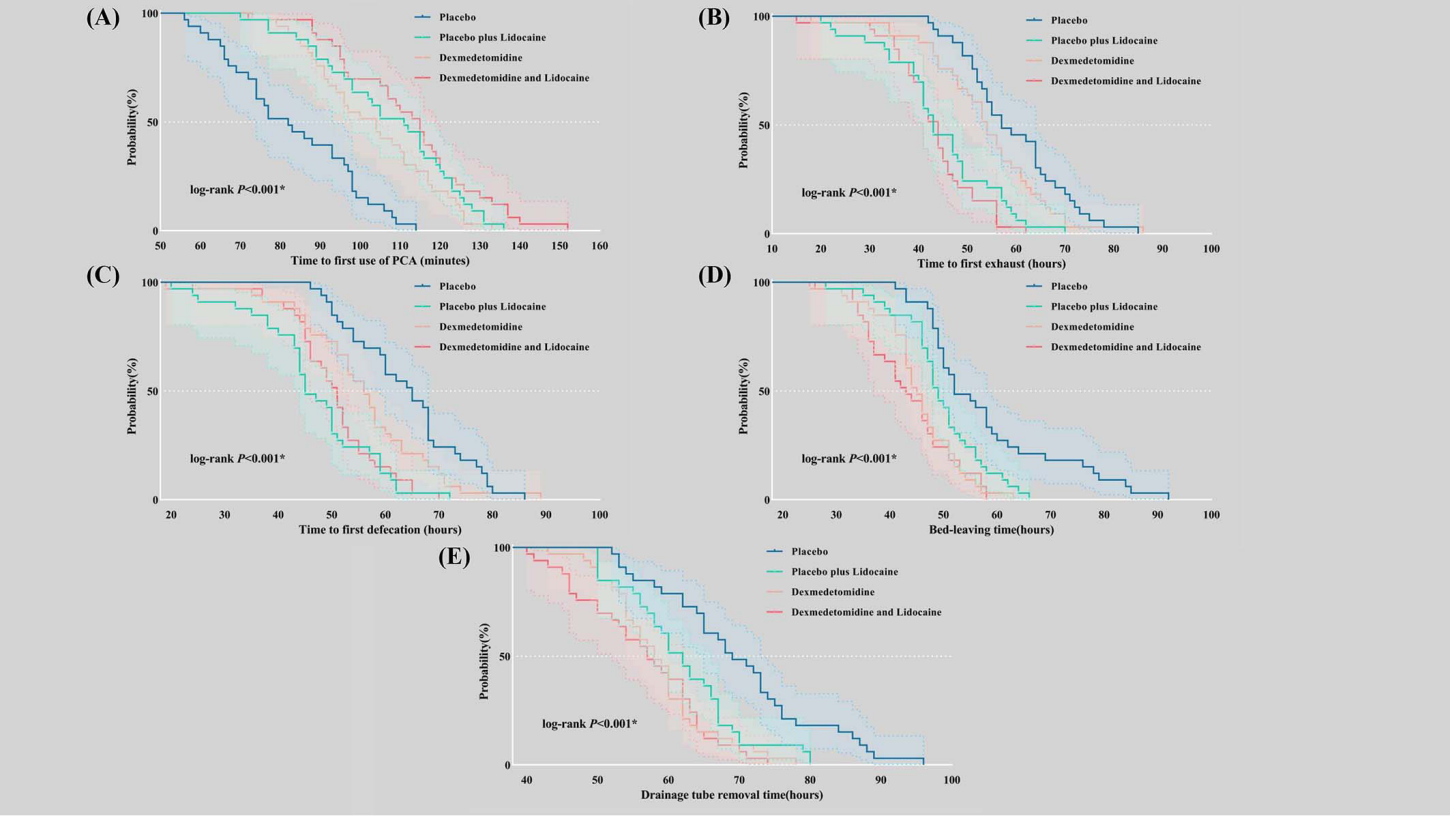
Kaplan−Meier curves were used to describe time-related outcomes during the postoperative period. **(A)** Time to first use of PCA; **(B)** Time to first exhaust; **(C)** Time to first defecation; **(D)** Bed-leaving time; **(E)** Drainage tube removal time. PCA, patient-controlled analgesia.

There were significant differences between the four groups concerning the time to the first PCA (*P*<0.001), exhaustion (*P*<0.001), defecation (*P*<0.001), bed-leaving (*P*<0.001), and drainage tube removal (*P*<0.001) (**Figures 4A–E**). The incidence of PPCs during hospitalization (*P*=0.804), the severity of PPCs according to the Clavien−Dindo classification (*P*=0.485), and the length of stay (*P*=0.630) did not differ significantly between groups (**Supplementary Table 1**).

#### 3.3.6 Short-term outcomes

No deaths occurred within 30 days after surgery in our study, and no patients were readmitted due to surgical complications (**Supplementary Table 1**).

### 3.4 Subgroup analysis of NETs

Subgroup analysis investigating the effect of intravenous Lido and Dex on differences in NETs between the perioperative and postoperative periods were conducted. The subgroup categories were based on age (≤ 60 years *vs*. > 60 years), sex (female *vs*. male), cancer stage (T1-2 *vs*. Tis), surgical type (lobectomy *vs*. nonlobectomy) and presence of PPCs (Yes *vs.* No). There was no interaction between grouping factors and subgroup classifications except the presence of PPCs (*P*=0.003). (**Supplementary Table 2**).

### 3.5 Multivariable linear regression analysis of NETs

The effects of tumor metastasis biomarkers, inflammatory factors, and cellular immune function on NETs were explored. There was a positive correlation between VEGF-α, MMP-3, MMP-9, and NETs. IL-6 was positively correlated with MPO, while CD4+ T cells were negatively correlated with H3Cit (**Supplementary Table 3**, **Supplementary Figure 1**).

### 3.6 Exploring the correlation between NETs and clinical outcomes

Postoperative MPO levels were positively correlated with the incidence of PPCs (β=0.392, *P*<0.001). MPO and H3Cit were positively correlated with active VAS scores at POD 1 (β=0.217, *P*=0.014; β=0.216, *P*=0.015, respectively) and negatively correlated with QoR-40 scores at POD 1 (β=-0.226, *P*=0.011; β=-0.177, *P*=0.047, respectively). MPO and H3Cit were positively correlated with the time to first exhaust (β=0.363, *P*<0.001; β=0.298, *P*<0.001, respectively) and defecation (β=0.328, *P*<0.001; β=0.256, *P*=0.004, respectively). The patients who suffered postoperative infectious adverse events had a more significant reduction in MPO values in the trial groups (-81.78 ± 13.54, -156.99.70 ± 13.92, -129.09 ± 16.45, and -172.00 ± 40.78 in Group C, L, D, and LD, *P*<0.001, respectively).

## 4 Discussion

In this single-center, double-blind, randomized trial, we evaluated the effect of intraoperative intravenous administration of Lido and Dex on perioperative serum levels of biomarkers of NETs (MPO and H3Cit) as well as other biomarkers contributing to invasion of cancer in early-stage NSCLC patients. H3Cit, as the critical biomarker of NET formation, predicts the risk of high mortality in patients with cancer (18). We selected 24 hours postoperatively to measure NET biomarkers because previous studies have shown that NETs and MPO formation occur at highest levels on the first postoperative day (12). Higher postoperative levels of NETs are associated with disease progression in cancer surgery (19). Previous studies have revealed that NETs could promote NSCLC metastasis through the NF-κB/NLRP3 inflammatory pathway (7) and facilitate metastasis of circulating lung carcinoma cells to the liver after surgery (8). Our results suggested that both Lido and Dex reduce the formation of NETs. The underlying mechanism might be that Lido and Dex inhibit the activation of the TLR2/NF-κB/NLRP3 pathway, high mobility group box-1 (HMGB-1), and granulocyte-colony stimulating factor (G-CSF) as suggested from the results of inflammation studies (20–22). HMGB-1 and G-CSF are crucial mediators in the initial formation of NETs (23). Another possible mechanism might be the activation by Dex of α2-adrenergic receptors expressed on neutrophils and the consequent desensitization of neutrophils to cytokine activation (24).

In the study by Hunter T et al., continuous perioperative intravenous Lido was shown to reduce NET formation directly (25). In contrast, the impact of Dex on NETs was not immediate (26). These results might explain why Dex alone did not reduce MPO and H3Cit levels as much as Lido alone. Evidence suggests that the intraoperative use of propofol rather than sevoflurane in breast cancer patients reduces the production of NETs, and the addition of Lido reduces NETs even more (13). Our study demonstrated that Lido and Dex could reduce the production of NETs despite the decreased usage of intraoperative propofol.

Lido has been proven to inhibit lung cancer growth through multiple mechanisms *in vitro* or *in vivo (* 27). Piegeler et al. (28) proved that Lido at clinically relevant concentrations diminished lung adenocarcinoma cell invasion and MMP-9 secretion by vitiated SRC-dependent inflammatory signaling conduction. Our trial revealed that either Lido or Dex decreased the serum levels of MMP-3 and MMP-9 after surgery, and their usage in combination significantly decreased MMP-3. In agreement with our speculation, decreased concentrations of both MMP-3 and MMP-9 were observed, seemingly because the suppression of MMP-3 and TNF-α levels led to a decrease in MMP-9 (29). Favorably for us, Galoș et al. (13) demonstrated that intravenous Lido could promote the postsurgical decrease in MMP-3 expression and NET formation in breast cancer patients. Also in line with our results, Zhang et al. (12) confirmed that intravenous Lido infusion reduced the appearance of NETs and MPO-DNA complexes in blood on POD 1 and POD 3 in pancreatic cancer patients. Furthermore, Zhang’s results ultimately suggested no effects of intravenous Lido perioperatively on the patients’ overall survival (OS) and disease-free survival (DFS). In our findings, Dex did not decrease the level of VEGF-α, but Lido did.

Recent studies have proven that Dex could paradoxically impact lung cancer. Lavon et al. (30) found that Dex increased tumor cell retention and growth of metastases of Lewis lung carcinoma in BALB/c mice. A similar perspective could be seen in Wang’s study (31). However, other investigations proved that treatment with clonidine, an α2- adrenoreceptor agonist, was not associated with shorter DFS or OS after lung cancer surgery (32). In our study, Dex reduced the formation of NETs and the expression of MMP-3 and MMP-9, which might retard the EMT of cancer clinically. However, without long-term follow-up, it is not known whether there was any effect on the patients’ DFS or OS.

The change in IL-6 expression has been proven to be used as an indicator of postoperative stress level (33). It is closely related to the occurrence of cancer-related pain and prognosis in NSCLC patients (34). Our results suggested that intravenous infusion of Lido and Dex could significantly reduce the serum level of IL-6.

Tumor cells rely on immune evasion to augment survival and often benefit from postoperative immunosuppressive states, which are closely related to tumor metastasis (35). Our trial implied that Lido and Dex had a significant impact on the equilibrium of the IFN-γ:IL4 ratio, which was a substitute indicator of the Th1:Th2 ratio. This equilibrium has been recognized as influencing the preservation of cellular immune function and is essential in suppressing tumor metastasis (36). Lido and Dex also increased the CD4+ T-cell ratio, but they did not significantly affect the equilibrium of CD4+ and CD8+ T cells, a result which is similar to the findings of another study (37). The lymphocyte count generally decreased significantly after surgery, and this trend was mitigated by Lido and Dex in this study.

Reducing suppression of immune function in the perioperative period curtails tumor metastasis, and NK cells can eradicate circulating cells that act as critical inhibitors of metastatic spread (38). Previous studies have shown that the use of opioids decreased NK cell function in patients one day after surgery (39). The stability of IFN-γ levels is closely related to the ordinary operation of NK cells (40). The results of our trial showed that Lido and Dex could preserve more NK cells, enhance IFN-γ status, and increase IL-2 levels postoperatively, which was strongly associated with better survival of NSCLC patients (41). This effect might be attributed to lower levels of stress, less consumption of opioids (42) and increased expression of NKG2D receptors on NK cells (37, 43, 44). Our study revealed that Lido and Dex could reduce the dosage of opioids required perioperatively. Studies have shown that intraoperative opioid exposure is associated with poor OS in early-stage NSCLC patients (45), which implies that Lido and Dex might have the ability to improve OS in NSCLC patients.

Our results suggested that in the Lido and Dex groups, the quality of recovery was briefly improved on POD 1 and POD 2, and the VAS scores at rest and at activity were both ephemerally ameliorated on POD 1 as observed in previous trials (12, 46). Once Lido and Dex were discontinued, the pain relief effect disappeared quickly, and the improvement in recovery quality lasted only up to 24 hours after discontinuation. Postoperative pumping of Lido and Dex could not only replace the analgesic effects of opioids but also reduce the frequency and total consumption of postoperative opioids. The opioid-sparing effect observed in our study could bring many benefits for NSCLC patients, such as early recovery of the gastrointestinal tract, early bed-leaving, and early drainage tube removal, without significantly increasing the occurrence of adverse events; this has been indicated by the results of a previous study as well (47).

A previous study proved that the 5-year overall and relapse-free survival rates were significantly worse in NSCLC patients with PPCs than in those without PPCs (48). Our results suggested that the trial group showed a better reduction in NETs in patients who developed PPCs. Although we did not observe differences in PPCs between groups, the initial confirmation of a positive correlation between higher levels of postoperative MPO and PPCs may provide some clues for future studies. In contrast to the results of our study, a previous study showed that intraoperative lidocaine infusion reduces postoperative PPCs (49). The reason for the difference in results may be the different definition of PPCs and the sample size of the study. Like a previous study (50), meaningful results regarding all-cause mortality and readmission rates were not observed in our study due to complications within 30 days after surgery.

While exploring the effects of tumor metastasis biomarkers, inflammatory factors, and cellular immunity on NETs, we found that VEGF-α, MMP-3, and MMP-9 were positively correlated with NETs, which is mechanistically plausible based on previous studies (51, 52). A positive correlation between IL-6 and NETs has been demonstrated in many studies (53, 54). Previous studies have shown that elevated NETs can increase Treg cells (55), and Treg cells decrease the proportion of CD4+ T cells, thereby weakening the body’s antitumor cellular immune function (56). Our results show a negative correlation between CD4+ T cells and NETs in agreement with these studies.

Above all, we hypothesized that adding Lido and Dex to general anesthesia may affect the metastasis and recurrence of NSCLC. This speculation is worthy of further evaluation in a large randomized clinical trial. At the same time, whether NETs can be used as an independent risk factor to predict the prognosis of NSCLC patients, especially those with concomitant postoperative infectious complications or PPCs, deserves further investigation.

## 5 Limitations

Our study has some limitations. First, the direct effects of Lido and Dex on tumor cells or tissues were not tested, and we have no long-term follow-up data to prove whether Lido and Dex have impacts on the OS or DFS of NSCLC patients. Second, blood samples were not collected at the end of surgery and tested. In addition, intraoperative tumor metastasis biomarkers and stress levels were not evaluated. Meanwhile, the impact of NETs and other tumor metastasis biomarkers on the survival of NSCLC patients remains unknown, and the current evidence is mainly derived from basic experiments. Also, the upstream factor VEGF-α, i.e., the hypoxia-induced factor, was not assessed.

## 6 Conclusions

This trial is the first study designed to investigate the effect of the perioperative application of Lido and Dex on NETs in NSCLC patients. Perioperative continuous intravenous infusion of Lido and Dex significantly reduced the production of NETs (MPO and H3Cit) and the expression of tumor metastasis biomarkers (VEGF-α, MMP-3, and MMP-9) in the peripheral blood of NSCLC patients. At the same time, Lido and Dex could also decrease inflammation, protect cellular immune function, reduce pain and opioid consumption, and improve the quality of postoperative recovery in NSCLC patients. It would be fruitful to pursue further research about whether the intravenous infusion of Lido and Dex can affect metastasis and recurrence in patients with NSCLC to improve the prognosis of such patients.

## Data availability statement

The original contributions presented in the study are included in the article/**Supplementary Material**. Further inquiries can be directed to the corresponding author.

## Ethics statement

The studies involving human participants were reviewed and approved by Ethics Committee of the Affiliated Hospital of Xuzhou Medical University. The patients/participants provided their written informed consent to participate in this study.

## Author contributions

BR, MC and CL contributed equally to this work and shared first authorship. They worked on the generation of article ideas, the elaboration of experimental protocols, the implementation of experiments and the process of writing articles. HZ, JYZ, WC and JS were involved in the collection of trial data and clinical samples. JWZ, TL, and RW were involved in the statistical processing of the experimental data and the collection of trial data and clinical samples. ZW was the general manager of the experiment and was involved in the development of the test protocol, the revision of the article and the supervision of the quality of the implementation of the experiment and the implementation process. All authors contributed to the article and approved the submitted version.

## Supplementary material

Supplementary Figure 1 Linear regression (A) with MPO as the dependent variable; (B) with H3Cit as the dependent variable. Dif., difference; MPO, myeloperoxidase; H3Cit, citrullinated histone 3; VEGF-A, vascular endothelial growth factor-A; MMP-3, matrix metalloproteinase-3; MMP-9, matrix metalloproteinase-9.
