# Supplementary material for: Perioperative lidocaine and dexmedetomidine intravenous infusion reduce the serum levels of NETs and biomarkers of tumor metastasis in lung cancer patients: A prospective, single-center, double-blinded, randomized clinical trial
Source: Front Oncol. 2023 Feb 24;13:1101449. doi: 10.3389/fonc.2023.1101449 (PMC10003334; doi:10.3389/fonc.2023.1101449)
Supplement: Supplementary file 1 [file DataSheet_1.docx]

**Supplementary Table 1. Other Secondary Outcomes and Short-term Outcomes**

| Variables | Group C (n=33) | Group L (n=33) | Group D (n=33) | Group LD (n=33) | *P*-value | Effect size |
| --- | --- | --- | --- | --- | --- | --- |
| **Other Secondary Outcomes** |  |  |  |  |  |  |
| Sufentanil consumption in 3 days postoperatively (μg) | 116.91±16.67 | 87.55±17.61† | 93.97±15.63† | 80.94±17.80†§ | <0.001* | 0.397 |
| VAS scores at rest |  |  |  |  |  |  |
| preoperative | 1(0.5-2) | 1(0-1.5) | 1(1-2) | 2(0-2) | 0.135 | 0.042 |
| POD1 | 4(3-6)Δ | 3(2-4)†Δ | 3(1.5-4)†Δ | 3(2-4)†Δ | <0.001* | 0.157 |
| POD2 | 4(2-5)Δ# | 3(2-4)Δ | 3(2-4)Δ# | 3(2-4.5)Δ | 0.864 | 0.006 |
| POD3 | 1(1-2)#¶ | 1(0-2) #¶ | 1(0-2)#¶ | 1(0-2)#¶ | 0.295 | 0.028 |
| VAS scores at activity |  |  |  |  |  |  |
| preoperative | 2(1-3) | 1(1-2) | 2(1-2) | 2(1-3) | 0.496 | 0.018 |
| POD1 | 5(4-6.5)Δ | 3(2-4.5)†Δ | 3(2-5)†Δ | 3(2-4)†Δ | <0.001* | 0.216 |
| POD2 | 4(3-6)Δ# | 4(2-5)Δ# | 3(2-4.5)Δ | 3(2-4.5)Δ | 0.035* | 0.065 |
| POD3 | 2(1-3)#¶ | 2(1-2)#¶ | 1(1-2)#¶ | 1(1-2)#¶ | 0.069 | 0.054 |
| Global QoR-40 difference |  |  |  |  |  |  |
| preoperative | 181.30±3.95 | 181.61±4.00 | 183.18±3.82 | 182.09±3.64 | 0.216 | 0.034 |
| POD1 | 163.33±7.71Δ | 171.82±5.68†Δ | 171.52±6.10†Δ | 172.18±3.51†Δ | <0.001* | 0.284 |
| POD2 | 168.03±8.39Δ# | 176.73±6.26†Δ# | 176.42±6.07†Δ# | 176.88±3.81†Δ# | <0.001* | 0.265 |
| POD3 | 177.30±4.08Δ#¶ | 177.52±4.71Δ# | 179.21±4.20Δ#¶ | 178.03±3.94Δ# | 0.264 | 0.030 |
| Time to the first use of PCA (minutes) | 83.09±16.67 | 107.03±17.64† | 103.00±15.91† | 112.09±17.81†§ | <0.001* |  |
| Time to the first exhaust (hours) | 59.55±10.69 | 44.27±11.71† | 53.55±12.67‡ | 43.09±9.09†§ | <0.001* |  |
| Time to the first defecation (hours) | 63.85±10.76 | 46.64±11.57† | 55.61±13.02†‡ | 50.24±9.05†§ | <0.001* |  |
| Bed-leaving time (hours) | 58.03±13.31 | 49.48±8.22† | 45.24±7.73†‡ | 43.45±8.36†‡ | <0.001* |  |
| Drainage tube removal time (hours) | 70.00±11.49 | 61.97±8.40† | 58.58±7.61† | 56.52±9.06†‡ | <0.001* |  |
| Incidence of postoperative pulmonary complications during hospitalization | 14(42.4) | 11(33.3) | 12(36.4) | 10(30.3) | 0.804 |  |
| Severity of pulmonary complications according to Clavien-Dindo classification | 0(0,1) | 0(0,1) | 0(0,1) | 0(0,1) | 0.485 |  |
| Length of hospital stay (days) | 6(5-6) | 5(5-6) | 5(5-6) | 6(5-6) | 0.630 |  |
| **Short-term outcomes** |  |  |  |  |  |  |
| Mortality within 30 days after surgery | 0 | 0 | 0 | 0 | 1.000 |  |
| Readmission rate within 30 days after surgery | 0 | 0 | 0 | 0 | 1.000 |  |

The data were the mean ± SD, n (%), or median (interquartile range). Notes: Group C, placebo group; Group L, placebo plus lidocaine group; Group L, dexmedetomidine plus placebo group; Group LD, dexmedetomidine plus lidocaine group. Abbreviations: VAS, Visual analog scale; QoR-40, Quality of Recovery 40; POD, Postoperative day; PCA, patient-controlled analgesia. A generalized estimating equation (GEE) was applied for repeated measures, choosing robust estimation as a covariance matrix. An unstructured working correlation matrix was performed in GEE. Bonferroni correction was applied during multiple comparisons. The effect size was expressed as partial η squared. **P*<0.05 was statistically significant.

†Compared with the placebo group(C), the difference was statistically significant.

‡Compared with the placebo plus lidocaine group (L), the difference was statistically significant.

§Compared with the dexmedetomidine plus placebo group (D), the difference was statistically significant.

ΔCompared with preoperative, the difference was statistically significant.

#Compared with POD1, the difference was statistically significant.

¶Compared with POD2, the difference was statistically significant.
