# Supplementary material for: Perioperative lidocaine and dexmedetomidine intravenous infusion reduce the serum levels of NETs and biomarkers of tumor metastasis in lung cancer patients: A prospective, single-center, double-blinded, randomized clinical trial
Source: Front Oncol. 2023 Feb 24;13:1101449. doi: 10.3389/fonc.2023.1101449 (PMC10003334; doi:10.3389/fonc.2023.1101449)
Supplement: Supplementary file 2 [file DataSheet_2.docx]

**Supplementary Table 2. Subgroups analysis of NETs.**

| Variables | *n* | Group C (n=33) | Group L (n=33) | Group D (n=33) | Group LD (n=33) | *P*-value for interaction |
| --- | --- | --- | --- | --- | --- | --- |
| MPO (U/ml) Dif | 132 | -106.51±25.44 | -197.08±34.01 | -137.37±32.41 | -189.45±33.73 |  |
| Subgroups |  |  |  |  |  |  |
| Age |  |  |  |  |  | 0.171 |
| =<60 | 66 | -102.69±27.56 | -203.83±30.45 | -131.83±33.24 | -197.37±33.48 |  |
| >60 | 66 | -109.69±23.86 | -190.73±36.94 | -147.06±29.77 | -183.62±33.60 |  |
| Sex |  |  |  |  |  | 0.871 |
| Female | 67 | -102.73±26.10 | -191.12±36.74 | -138.03±28.99 | -186.60±34.21 |  |
| Male | 65 | -110.52±24.93 | -202.69±31.48 | -136.75±36.22 | -192.14±34.10 |  |
| Cancer stage |  |  |  |  |  | 0.759 |
| Tis | 32 | -117.95±22.60 | -203.48±31.22 | -141.01±36.27 | -205.26±28.46 |  |
| I or II | 100 | -102.85±25.62 | -195.03±35.32 | -136.56±32.19 | -182.58±34.07 |  |
| Surgical type |  |  |  |  |  | 0.606 |
| Lobectomy | 51 | -101.59±28.83 | -193.08±36.17 | -137.76±32.55 | -180.67±34.42 |  |
| Non-Lobectomy | 81 | -111.73±20.93 | -205.08±29.45 | -136.46±33.81 | -201.37±29.92 |  |
| Presence of postoperative pulmonary complications |  |  |  |  |  | 0.003 |
| Yes | 47 | -81.78±13.54 | -156.99±13.92 | -129.09±16.45 | -172.00±40.78 |  |
| No | 85 | -124.73±13.87 | -217.12±20.41 | -142.10±38.30 | -197.04±27.86 |  |
| H3Cit (ng/ml) Dif | 132 | -24.73±7.65 | -49.51±9.11 | -34.80±10.37 | -51.82±8.98 |  |
| Subgroups |  |  |  |  |  |  |
| Age |  |  |  |  |  | 0.389 |
| =<60 | 66 | -24.12±7.73 | -48.73±8.22 | -34.30±9.83 | -54.77±10.74 |  |
| >60 | 66 | -25.23±7.77 | -50.25±10.07 | -35.67±11.65 | -49.64±6.95 |  |
| Sex |  |  |  |  |  | 0.731 |
| Female | 67 | -25.66±7.14 | -48.65±8.61 | -33.44±11.01 | -52.17±8.90 |  |
| Male | 65 | -23.73±8.27 | -50.32±9.75 | -36.07±9.90 | -51.49±9.32 |  |
| Cancer stage |  |  |  |  |  | 0.481 |
| Tis | 32 | -26.31±7.66 | -45.67±7.99 | -31.42±10.44 | -48.75±8.39 |  |
| I or II | 100 | -24.22±7.73 | -50.74±9.24 | -35.55±10.40 | -53.15±9.07 |  |
| Surgical type |  |  |  |  |  | 0.448 |
| Lobectomy | 51 | -23.72±6.19 | -49.27±8.87 | -35.47±10.46 | -53.82±8.73 |  |
| Non-Lobectomy | 81 | -25.80±9.04 | -50.00±9.99 | -33.24±10.54 | -49.10±8.90 |  |
| Presence of postoperative pulmonary complications |  |  |  |  |  | 0.499 |
| Yes | 47 | -24.62±8.52 | -47.57±8.85 | -33.40±10.84 | -54.52±9.19 |  |
| No | 85 | -24.80±7.18 | -50.48±9.28 | -35.59±10.28 | -50.64±8.83 |  |

The data were the mean ± SD. Notes: Group C, placebo group; Group L, placebo plus lidocaine group; Group L, dexmedetomidine plus placebo group; Group LD, dexmedetomidine plus lidocaine group. Subgroup analysis investigating the effect of intravenous lidocaine and dexmedetomidine on differences of serum NETs levels between perioperative and postoperative, according to age (≤ 60 y *vs.* > 60 y), sex (female *vs.* male), cancer stage (T1-2 *vs.* Tis), surgical type (lobectomy *vs.* non-lobectomy) and presence of postoperative pulmonary complications (Yes *vs.* No). Abbreviations: Dif., differences; NETs, neutrophil extracellular traps; MPO, myeloperoxidase; H3Cit, citrullinated histone 3. Non-lobectomy includes segmentectomy and wedge resection. All differences were obtained by subtracting the preoperative level from the postoperative level. **P*<0.05 was statistically significant.
