# Supplementary material for: Perioperative lidocaine and dexmedetomidine intravenous infusion reduce the serum levels of NETs and biomarkers of tumor metastasis in lung cancer patients: A prospective, single-center, double-blinded, randomized clinical trial
Source: Front Oncol. 2023 Feb 24;13:1101449. doi: 10.3389/fonc.2023.1101449 (PMC10003334; doi:10.3389/fonc.2023.1101449)
Supplement: Supplementary file 3 [file DataSheet_3.docx]

**Supplementary Table 3. Multivariable linear regression analysis of NETs.**

| **Dependent variable** | **Independent variable** | **β-coefficient and 95%CI** | **Standardized β-coefficient** | ***P*-value** | **VIF** | **Adjusted-R^2^** |
| --- | --- | --- | --- | --- | --- | --- |
| MPO Dif | Intercept | -84.652 (-126.259, -43.045) |  |  |  | 0.509 |
|  | VEGF-α Dif | 0.333 (0.042, 0.623) | 0.189 | 0.025* | 1.847 |  |
|  | MMP-3 Dif | 0.512 (0.149, 0.875) | 0.221 | 0.006* | 1.665 |  |
|  | MMP-9 Dif | 6.012 (2.858, 9.166) | 0.321 | <0.001* | 1.928 |  |
|  | IL-6 Dif | 0.177 (0.028, 0.327) | 0.167 | 0.021* | 1.359 |  |
| H3Cit Dif | Intercept | -16.255 (-25.429, -7.081) |  |  |  | 0.549 |
|  | VEGF-α Dif | 0.162 (0.082, 0.243) | 0.315 | <0.001* | 1.814 |  |
|  | MMP-3 Dif | 0.163 (0.063, 0.264) | 0.241 | 0.002* | 1.632 |  |
|  | MMP-9 Dif | 1.238 (0.363, 2.112) | 0.226 | 0.006* | 1.889 |  |
|  | CD4+T cells Dif | -2.082 (-3.402, -0.763) | -0.194 | 0.002* | 1.124 |  |

Abbreviations: Dif., differences; NETs, neutrophil extracellular traps; MPO, myeloperoxidase; H3Cit, citrullinated histone 3; VEGF-α, vascular endothelial growth factor-α; MMP-3, matrix metalloproteinase-3; MMP-9, matrix metalloproteinase-9; VIF, variance inflation factor. Tumor metastasis biomarkers, inflammatory factors, and cellular immune function were included as independent variables, and NETs were taken as dependent variables in the multifactorial regression. The stepwise method was chosen when the probability corresponding to the F value of the independent variable was included if <0.05 and excluded if >0.10. **P*<0.05 was statistically significant. All differences were obtained by subtracting the preoperative level from the postoperative level.
