# Supplementary figures and images for: Perioperative lidocaine and dexmedetomidine intravenous infusion reduce the serum levels of NETs and biomarkers of tumor metastasis in lung cancer patients: A prospective, single-center, double-blinded, randomized clinical trial
Source: Front Oncol. 2023 Feb 24;13:1101449. doi: 10.3389/fonc.2023.1101449 (PMC10003334; doi:10.3389/fonc.2023.1101449)

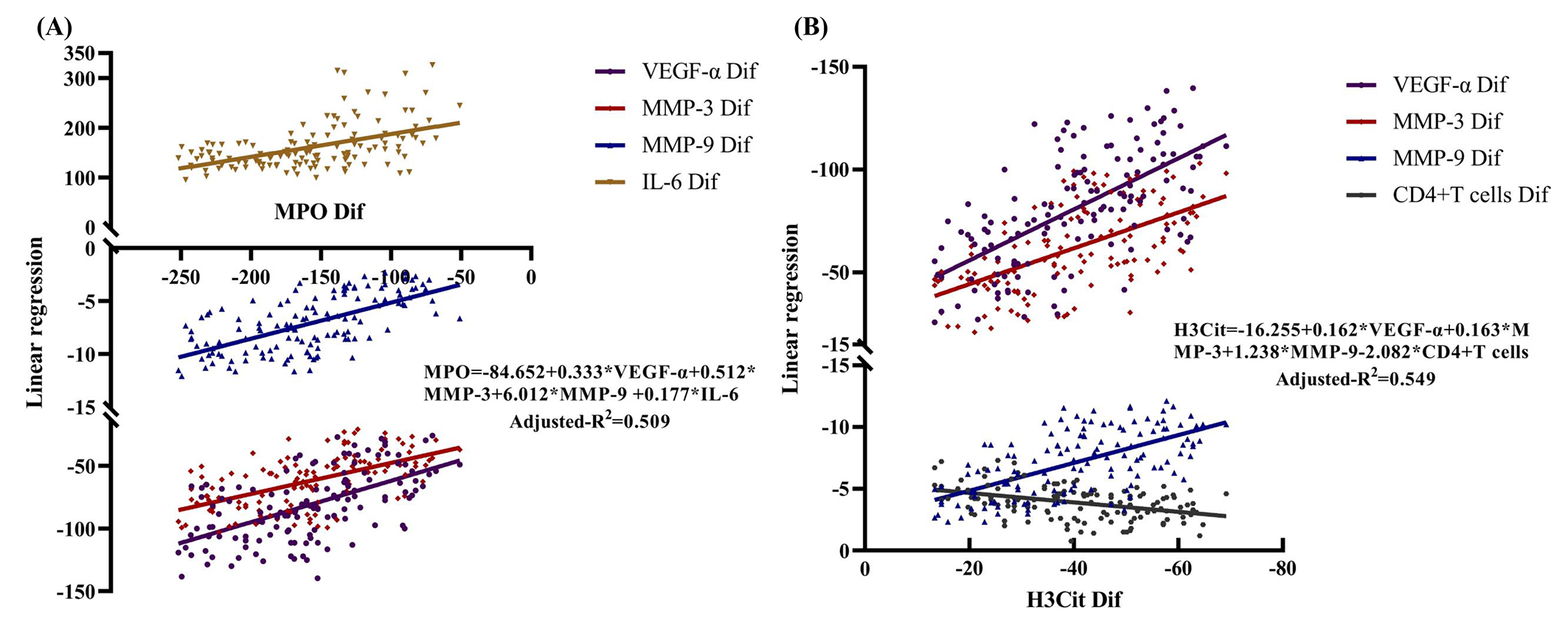

Supplement: Supplementary Figure 1 — Linear regression (A) with MPO as the dependent variable; (B) with H3Cit as the dependent variable. Dif., difference; MPO, myeloperoxidase; H3Cit, citrullinated histone 3; VEGF-A, vascular endothelial growth factor-A; MMP-3, matrix metalloproteinase-3; MMP-9, matrix metalloproteinase-9. [file Image_1.jpeg]
